# Role of the unique N-terminal domain of CtBP2 in determining the subcellular localisation of CtBP family proteins

**DOI:** 10.1186/1471-2121-7-35

**Published:** 2006-09-25

**Authors:** Lee M Bergman, Laila Morris, Matthew Darley, Alexander H Mirnezami, Samal C Gunatilake, Jeremy P Blaydes

**Affiliations:** 1 Cancer Sciences Division, School of Medicine, University of Southampton, Southampton SO16 6YD, UK

## Abstract

**Background:**

CtBP1 and CtBP2 are transcriptional co-repressors that modulate the activity of a large number of transcriptional repressors via the recruitment of chromatin modifiers. Many CtBP-regulated proteins are involved in pathways associated with tumorigenesis, including TGF-β and Wnt signalling pathways and cell cycle regulators such as RB/p130 and HDM2, as well as adenovirus E1A. CtBP1 and CtBP2 are highly similar proteins, although evidence is emerging that their activity can be differentially regulated, particularly through the control of their subcellular localisation. CtBP2s from diverse species contain a unique N-terminus, absent in CtBP1 that plays a key role in controlling the nuclear-cytoplasmic distribution of the protein.

**Results:**

Here we show that amino acids (a.a.) 4–14 of CtBP2 direct CtBP2 into an almost exclusively nuclear distribution in cell lines of diverse origins. Whilst this sequence contains similarity to known nuclear localisation motifs, it cannot drive nuclear localisation of a heterologous protein, but rather has been shown to function as a p300 acetyltransferase-dependent nuclear retention sequence. Here we define the region of CtBP2 required to co-operate with a.a. 4–14 to promote CtBP2 nuclear accumulation as being within a.a. 1–119. In addition, we show that a.a. 120–445 of CtBP2 can also promote CtBP2 nuclear accumulation, independently of a.a. 4–14. Finally, CtBP1 and CtBP2 can form heterodimers, and we show that the interaction with CtBP2 is one mechanism whereby CtBP1 can be recruited to the nucleus.

**Conclusion:**

Together, these findings represent key distinctions in the regulation of the functions of CtBP family members that may have important implications as to their roles in development, and cell differentiation and survival.

## Background

CtBP proteins were originally identified as C-terminal binding proteins of type 2/5 adenovirus E1A proteins [1]. They function primarily in the nucleus as transcriptional co-repressors, modulating the activity of a large number of transcriptional repressors via recruitment of chromatin modifiers such as histone deacetylases, histone methyltransferases and polycomb proteins [2-4], and sequestration of histone acetyltransferases [5]. CtBP proteins also play a role in the cytoplasm in regulating mitotic Golgi membrane fissioning [6,7], and also associate with centrosomes during mitosis [8]. CtBP proteins have been implicated in tumorigenesis, as their interaction with the C-terminus of E1A is essential for immortalisation of primary rodent cells, and also negatively regulates E1A-mediated transformation, tumorigenicity and metastasis [1,9,10]. In addition, many transcriptional repressors regulated by CtBPs are involved in pathways associated with tumorigenesis, including TGF-β and Wnt signalling pathways and cell cycle regulators such as RB/p130 and HDM2 [11-15]. Presumably, as a consequence of disruption of some of these critical functions, inhibition of CtBP expression in cancer cells can result in apoptosis [16]; reviewed in [17].

Humans possess two CtBP gene loci, *CTBP1 *and *CTBP2*. CtBP1 and CtBP2 proteins share 78% amino acid identity and 83% similarity [18]. Alternate promoter usage and gene splicing from the *CTBP2 *locus generates RIBEYE, a retina-specific CtBP2 variant [19]. The *CTBP1 *locus also similarly encodes a CtBP1 variant with an alternate N-terminus, variously described as CtBP3, BARS or CtBP1-S [20]. The primary protein products, CtBP1 and CtBP2, both contain a conserved N-terminal domain involved in the binding of transcription factors possessing a consensus PxDLS peptide motif, and a central dehydrogenase homology domain that has a number of functions, including dimerisation. CtBP1 and CtBP2 appear to function interchangeably, at least in terms of their role as transcriptional co-repressors, but evidence is emerging that they are subject to differential transcriptional and post-translational regulation (reviewed in [21]).

Control of subcellular localisation is emerging as an important mechanism whereby CtBP1 function is regulated. For example, phosphorylation of CtBP1 at Ser158 by p21-activated kinase 1 (PAK1) results in cytoplasmic localisation and inhibition of its corepressor activity under certain growth conditions [22]. Certain PxDLS-containing transcriptional repressors are able to recruit CtBP1 to the nucleus, such as Ets family member NET [23] and the tumour suppressor protein HIC1 [24]. CtBP1 is modified by sumoylation at K428, which, in conjunction with protein-protein interactions involving its C-terminal PDZ-binding domain [25,26], regulates its nuclear localisation [26]. CtBP2 lacks both this sumoylation site and the PDZ-binding domain, indicating that its subcellular localisation is likely to be regulated in a different manner to CtBP1. We therefore examined the primary sequence of CtBP2 to look for alternative sequence motifs that could be involved in the regulation of its localisation. This analysis identified a putative, evolutionarily conserved nuclear localisation signal (NLS), which has recently been shown to be functional in promoting the nuclear accumulation of CtBP2, [27,28] though it has been shown to function in nuclear retention, rather than nuclear import [27]. In this present study, we have undertaken a detailed analysis of the role played by this N-terminal sequence of CtBP2 in regulating CtBP protein localisation.

## Results

### Structure and phylogenetics of *CTBP *loci

**Figure 1:**
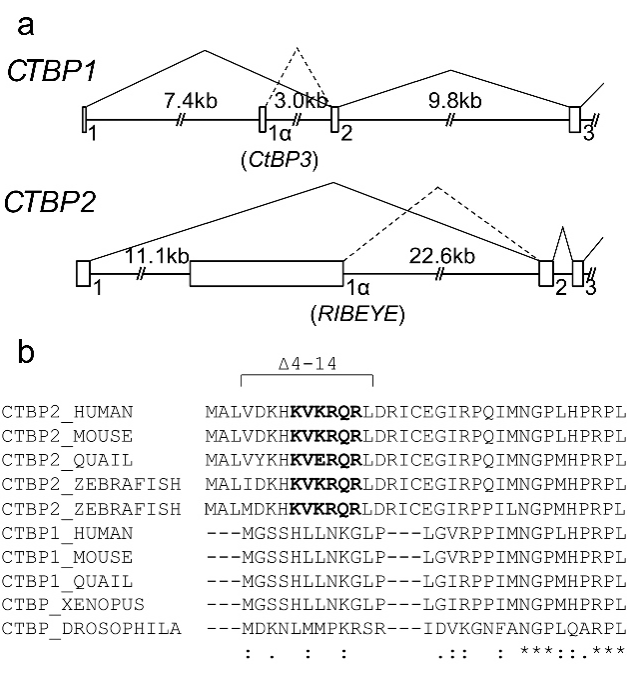
**CtBP gene structure and sequence comparison**. (**a**) Genomic structure of the 5' end of human *CTBP1 *and *CTBP2 *genes. Published cDNA sequences were compared to human genome sequences using BLAST analysis. Solid lines show splicing of the major *CTBP1 *and *CTBP2 *transcripts, and dotted lines indicate the alternate splicing to generate CtBP1-S and RIBEYE. (**b**) ClustalW alignment of CtBP sequences from multiple higher organisms. Putative nuclear localisation signals are highlighted in bold type. The residues deleted in the Δ4–14 constructs are marked. Zebrafish have two *ctbp2/ribeye *loci [36].

The CtBP2 protein sequence was subjected to an *in silico *search for potential nuclear localisation signals [29] and a potential NLS (KxKRQR) was identified at amino acids (a.a.) 8–13. Because this sequence is located within the non-conserved N-terminus of CtBP2, and because of the differential promoter usage and alternative splicing of the variant CtBP proteins, we first clarified the genomic structures of the 5' regions of the *CTBP1 *and *CTBP2 *loci (Fig. 1a). Fig. 1b shows the N-terminus of human CtBP2 and its homology to other known CtBP proteins. The putative NLS in human CtBP2 is conserved completely in mouse and zebrafish CtBP2, and contains a single amino acid substitution in quail CtBP2. CtBP1, as well as the single CtBP in *Drosophila *and *Xenopus*, do not contain this sequence, though *Drosophila *CtBP does contain a short lysine-arginine rich sequence (KRSR) that is not present in CtBP1 proteins. It is also interesting to note that a.a. 1–20 in CtBP2, including the putative NLS, is encoded by a short exon 1 that is located more than 30 kb upstream of the rest of the gene.

### Amino acids 4–14 of CtBP2 promote its localisation to the nucleus

**Figure 2:**
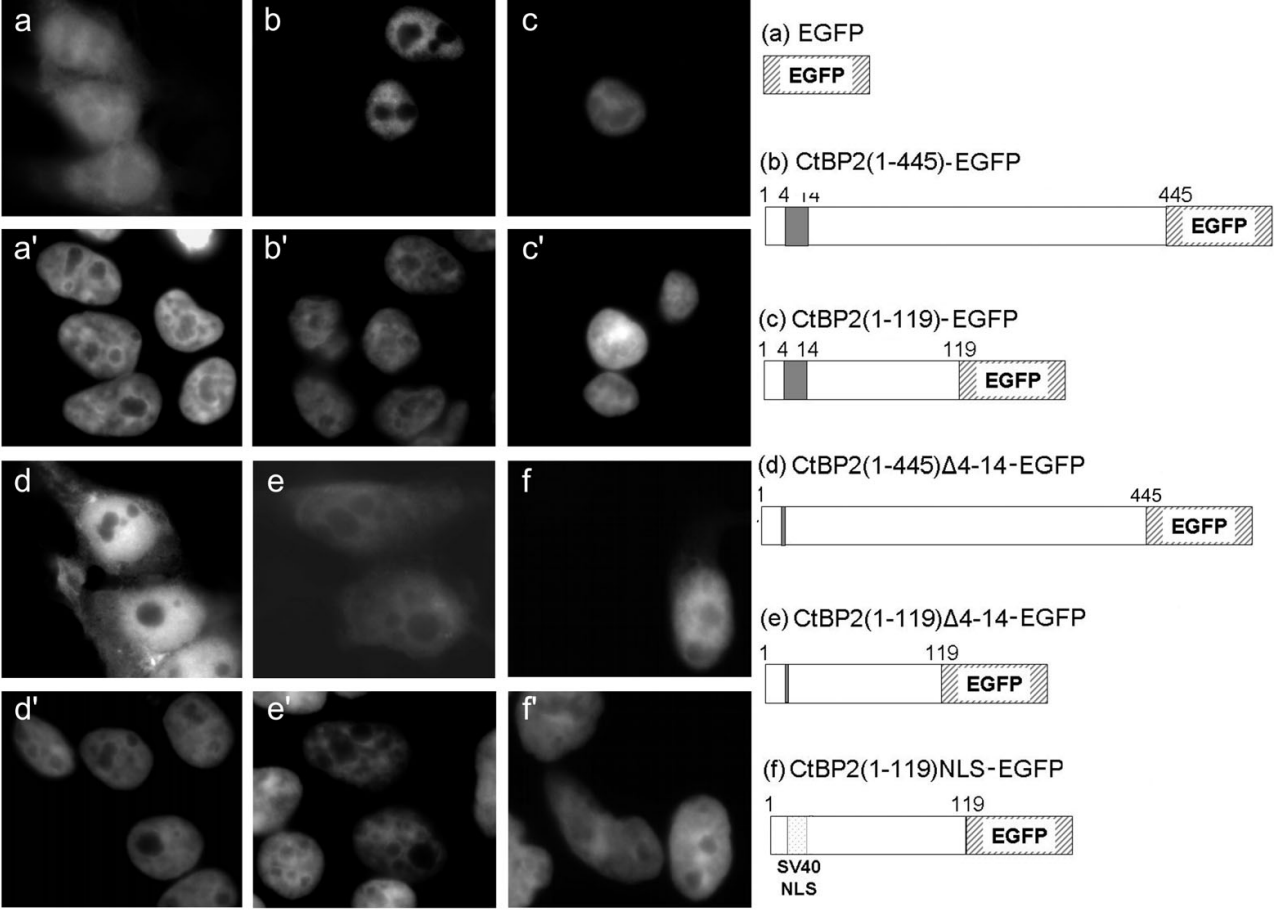
**Subcellular localisation of EGFP-tagged CtBP2 proteins in HEK 293 cells**. Images are as follows: empty pEGFP-N1 vector (**a**); CtBP2(1–445)-EGFP (**b**); CtBP2(1–119)-EGFP (**c**); CtBP2(1–445)Δ4–14-EGFP (**d**); CtBP2(1–119)Δ4–14-EGFP (**e**); CtBP2(1–119)NLS-EGFP (**f**). Corresponding DAPI nuclear counterstain (**a'-f'**). A schematic diagram of the CtBP2-EGFP constructs is shown.

In order to establish whether the unique N-terminal region of CtBP2 is important in determining CtBP2 subcellular distribution, we expressed various CtBP2-EGFP fusion proteins in HEK 293 cells (Fig. 2). 48 hours after transfection, cells were fixed and counterstained with DAPI, and analysed by fluorescence microscopy. Control, EGFP alone localised to the nucleus and cytoplasm (Fig. 2a). Both full-length CtBP2-EGFP (Fig. 2b) and a truncated version containing a.a. 8–13 and the N-terminal PxDLS-binding domain, CtBP2(1–119)-EGFP (Fig. 2c), were detectable exclusively in the nucleus. Deletion of eleven amino acids encompassing a.a. 8–13 in full length CtBP2 (CtBP2(1–445)Δ4–14-EGFP) resulted in a partial redistribution of the protein to the cytoplasm, although it was still predominantly nuclear (Fig. 2d). The Δ4–14 mutation was also made in the context of CtBP2(1–119)-EGFP (Fig. 2e), and resulted in a more pronounced redistribution to the cytoplasm compared to its effect on the full length protein, though again EGFP fluorescence was still detectable in the nucleus. Substitution of a.a. 4–14 with a *bona fide *NLS from SV40 large tumour antigen at the N-terminus of the truncated CtBP2 mutant (CtBP2(1–119)NLS-EGFP) also resulted in exclusive nuclear localisation (Fig. 2f). Thus, residues 4–14 of CtBP2 are important for maintaining its nuclear localisation, although other regions within CtBP2 protein are clearly also involved.

**Figure 3:**
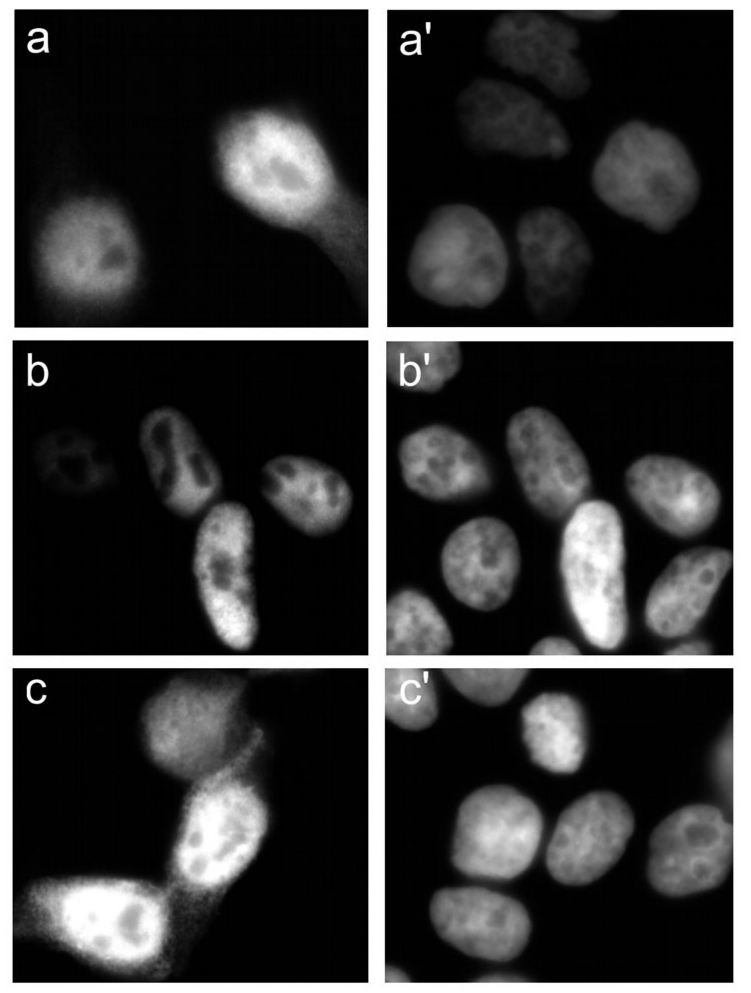
**Subcellular localisation of CtBP proteins in HEK 293 cells, using myc-his-tagged constructs**. Images are as follows: CtBP1(1–440)mh (**a**); CtBP2(1–445)mh (**b**); CtBP2(1–445)Δ4–14mh (**c**). Corresponding DAPI stains are shown in **a'-c'**.

To ensure that the above results were not affected by the presence of a large EGFP tag, we cloned various CtBP constructs into a vector containing a smaller myc-his tag (mh). Expressed proteins were detected using a 6xHis-specific primary antibody. A nuclear localisation in HEK 293 cells was confirmed for exogenous full-length CtBP2(1–445)mh (Fig. 3b). The deletion mutant (CtBP2(1–445)Δ4–14mh) showed a similar nuclear and cytoplasmic localisation to its corresponding EGFP fusion protein (Fig. 3c). We also cloned full length CtBP1 into this expression vector, in order to compare results with that of CtBP2, and with other studies. Consistent with previous studies on other cell lines, exogenous CtBP1(1–440)mh localises primarily to the nucleus, with some cytoplasmic staining (Fig. 3a).

**Figure 4:**
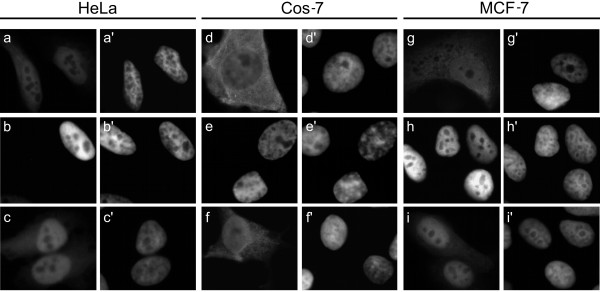
**Subcellular localisation of CtBP proteins in additional cell types**. Expression patterns of CtBP1(1–440)mh, CtBP2(1–445)mh and CtBP2(1–445)Δ4–14mh in HeLa cells, (**a,b,c **respectively), Cos-7 cells (**d,e,f **respectively) and MCF-7 cells (**g,h,i **respectively). Corresponding DAPI images are shown in **a'-i'**.

Cell type-specific differences have been observed in the degree of nuclear and cytoplasmic localisation of over-expressed CtBP1 [23,26,30]. We wanted to investigate the behaviour of CtBP2 following over-expression in different cell lines, and specifically whether it would still be localised to the nucleus in cells in which CtBP1 is cytoplasmic. As expected [26], CtBP1(1–440)mh is distributed in the both the nucleus and cytoplasm of HeLa cells, with staining being strongest in the nucleus (Fig. 4a). Similar to a previous report [23], we found that CtBP1(1–440)mh localises predominantly to the cytoplasm in over 60% of Cos-7 cells, with some cells showing a nuclear and cytoplasmic distribution (Fig. 4d). CtBP1(1–440)mh is nuclear and cytoplasmic in MCF-7 cells, similar to HeLa cells (Fig. 4g). CtBP2(1–445)mh localises exclusively to the nucleus of all three cell lines (Figs. 4b,e,h). In the absence of a.a. 4–14, CtBP2(1–445)Δ4–14mh remains primarily nuclear in all three cell lines, though with a clear increase in cytoplasmic staining similar to our findings in HEK 293 cells, (Figs. 4c,f,i). These experiments, particularly those with Cos-7 cells, confirm that the presence of a.a. 4–14 in CtBP2 confers upon it an almost exclusively nuclear distribution. This is in contrast to CtBP1, which shows cell type-dependent variations in its localisation.

### Binding of PxDLS-containing proteins is not required for the a.a. 4–14-independent nuclear localisation of CtBP2

**Figure 5:**
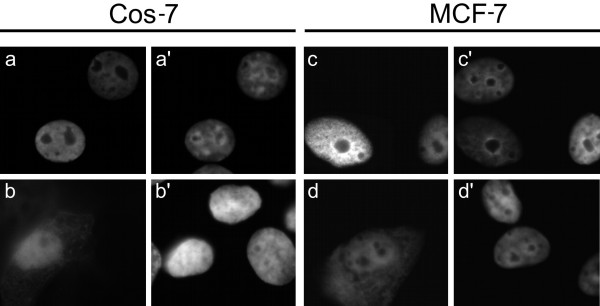
**Influence of PxDLS-containing proteins on CtBP2 subcellular localisation**. Cos-7 and MCF-7 cells were transfected with either CtBP2(1–445)V72Rmh (**a **and **c**, respectively) or CtBP2(1–445)Δ4–14V72Rmh (**b **and **d**, respectively). Corresponding DAPI stains are shown in **a'-d'**.

Our experiments show that even when a.a. 4–14 are absent, a large proportion of the CtBP2 still localises to the nucleus. As a previous study has shown that binding of a PxDLS-containing protein to CtBP1 promotes its nuclear localisation [25], we decided to investigate whether such an interaction may also drive the nuclear localisation of CtBP2. We introduced a point mutation (V72R) into the PxDLS-binding motif of the CtBP2(1–445)mh constructs to generate CtBP2(1–445)V72Rmh and CtBP2(1–445)Δ4–14V72Rmh. This mutation renders CtBPs defective in their interaction with PxDLS-containing proteins [31]. Full-length CtBP2(1–445)mh with the V72R mutation localises to the nucleus in both Cos-7 and MCF-7 cells (Fig. 5a,c). CtBP2(1–445)Δ4–14V72Rmh localises to both the nucleus and cytoplasm (Fig. 5b,d), with no further increase in cytoplasmic distribution compared to the CtBP2(1–445)Δ4–14mh protein (compare Figs. 5b,d with Figs. 4f,i). We therefore conclude that the a.a. 4–14-independent nuclear localisation of CtBP2 in these cells also occurs independently of its PxDLS binding ability.

### CtBP2 influences CtBP1 subcellular localisation

**Figure 6:**
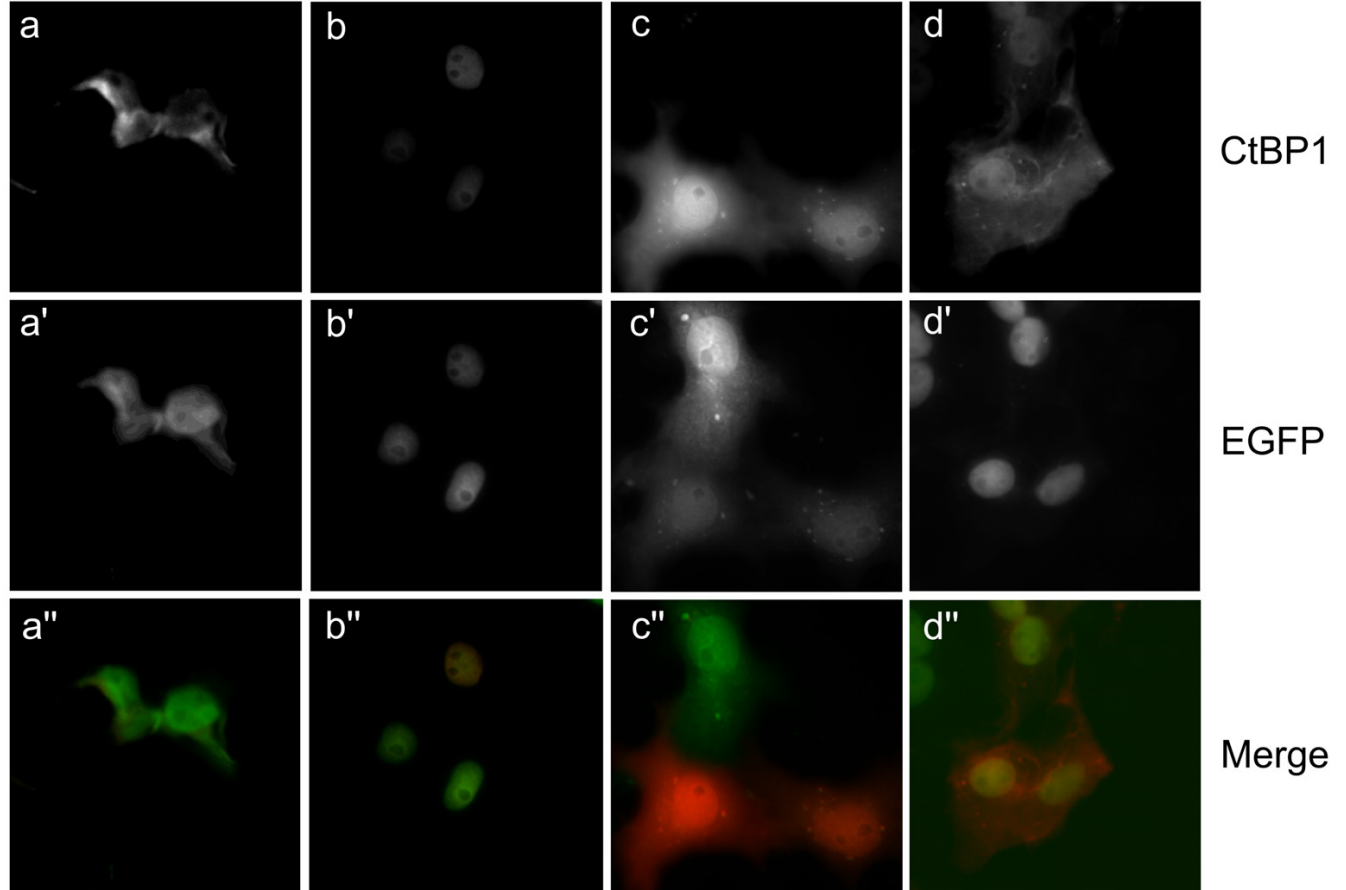
**Influence of CtBP2 on CtBP1 subcellular localisation**. Cos-7 cells were co-transfected with 0.2 μg CtBP1(1–440)mh plus 0.2 μg of either control pEGFP-N1 (**a,a',a"**), CtBP2(1–445)-EGFP (**b,b',b"**), CtBP2(1–445)Δ4–14-EGFP (**c,c',c"**); or CtBP2(1–119)-EGFP (**d,d',d"**). **a,b,c,d **show localisation of CtBP1(1–440)mh. **a',b',c',d' **show localisation of EGFP fusion proteins. **a",b",c",d" **are merges of the CtBP1 and EGFP images.

We, and others, have shown that the unique N-terminal region of CtBP2 is a major factor driving its accumulation in the nucleus. As CtBP1 lacks this sequence, the mechanisms determining its subcellular distribution remain an important question for understanding the regulation of its function. To this end, we asked whether heterodimerisation with CtBP2 might be able to recruit CtBP1 to the nucleus. To examine this, we analysed the effects of co-expressing both mhCtBP1 and various CtBP2-EGFP constructs in Cos-7 cells. CtBP1(1–440)mh, when transfected individually (Fig. 4d) or with EGFP-N1 control (Fig. 6a), is predominantly cytoplasmic. Co-expression of CtBP1(1–440)mh with CtBP2(1–445)-EGFP results in a striking relocalisation of CtBP1(1–440)mh to the nucleus in a high proportion of the cells (Fig. 6b). Quantification of this by counting stained cells showed over-expressed CtBP1(1–440)mh to be primarily cytoplasmic in 75% of the cells, with a mixed nuclear/cytoplasmic localisation in 25%. When co-transfected with CtBP2(1–445)-EGFP, this changes to 45% nuclear/cytoplasmic and 55% primarily nuclear. This effect is dependent on CtBP2 being correctly localised to the nucleus, as demonstrated by the effects of co-expressing CtBP1(1–440)mh and CtBP2(1–445)Δ4–14-EGFP (Fig. 6c). Finally, we demonstrate that the recruitment of CtBP1 to the nucleus by CtBP2 requires a.a. 120–445 of CtBP2, as co-expression of EGFP-CtBP2(1–119) does not alter the localisation of CtBP1(1–440)mh (Fig. 6d), and the two proteins fail to co-localise. As a.a. 120–445 contain the dimerisation domain, this finding is consistent with heterodimerisation with CtBP2 being a mechanism whereby CtBP1 can be recruited to the nucleus.

## Discussion

We show firstly that the deletion of eleven amino acids (Δ4–14) encompassing the putative NLS sequence KVKRQR at a.a. 8–13 of CtBP2 results in shift in the localisation of detectable CtBP2 from exclusively nuclear, to nuclear and partly cytoplasmic. This effect was observed in a number of cell lines of diverse origin. These initial findings confirm a recently published study which identified a role for a.a. 1–21 in the nuclear localisation and retention of CtBP2 [27], as well as another study which was published whilst this manuscript was being submitted [28]. Our data further localises the critical sequence elements to a.a. 4–14. One of the cell lines, Cos-7, was chosen because a previous study had shown that transfected CtBP1 primarily localises to the cytoplasm in these cells, making them a useful experimental model [23]. Amino acids 4–14 also direct nuclear accumulation of CtBP2 in Cos-7 cells. Therefore, whatever the mechanism that underlies the cytoplasmic localisation of CtBP1 expressed in Cos-7 cells, it does not affect the ability of a.a. 4–14 to localise CtBP2 to the nucleus. As a primary function of CtBP proteins is as nuclear transcriptional co-repressors, this sequence in CtBP2 is likely to play a key role in maintaining nuclear CtBP activity in cells where CtBP2 is expressed.

Before considering the role of a.a. 4–14 further, it is important to note that we, as well as Zhao *et al *[27] who examined the effects of an a.a. 1–21 deletion, observed that CtBP2 with these N-terminal sequences deleted still retained a predominantly nuclear localisation, with only a partial redistribution to the cytoplasm. Interestingly, Verger *et al *[28] found than a CtBP2Δ1–25 mutant localised almost exclusively in the cytoplasm in Cos-1 cells. This different result could be due to the slightly larger deletion that they used, the cell type, or experimental differences. However, our experiments clearly show that domains other than a.a. 4–14 can be important in defining the nuclear-cytoplasmic distribution of CtBP2. These could potentially become important under conditions whereby the N-terminal sequences of CtBP2 might be masked, such as following binding of another protein or through post-translational modification. As the localisation of CtBP2(1–445)Δ4–14mh in all four cell lines closely resembled that of transfected CtBP1 in HEK 293, HeLa and MCF-7 cells it is possible this a.a. 4–14-independent nuclear localisation occurs through the same mechanisms that regulate CtBP1. In studies on Cos-7 cells, Criqui-Philipe *et al *[23] showed that CtBP1 could be recruited to the nucleus through an association with PxDLS containing transcription factors. Structural studies on rat CtBP1-S (BARS) have characterised the PxDLS-binding interface, and identified mutations (e.g. V55R) that disrupt CtBP-binding to the C-terminal domain of E1A. We therefore examined the effects of generating the CtBP2 equivalent of the CtBP1-SV55R mutation. Our finding that this V72R mutation of CtBP2(1–445)Δ4–14mh does not affect its subcellular localisation excludes the PxDLS-binding interface as the major determinant for the a.a. 4–14-independent localisation of CtBP2 to the nucleus in these experiments. The N-terminal 119 a.a of CtBP2 is able to engage in other protein-protein interactions through less well-defined interfaces, e.g. [12], and a role for these interactions in CtBP2 subcellular distribution cannot yet be excluded.

Our experiments showed that CtBP2(1–119)Δ4–14mh has a markedly more cytoplasmic distribution than CtBP2(1–445)Δ4–14mh. This identifies a.a. 120–445 as having a role in nuclear localisation. When compared to domains in this region of CtBP1 with a known role in subcellular localisation, CtBP2 lacks the PDZ binding motif present at the extreme C-terminus of CtBP1 [25], as well as the equivalent of the sumoylation site at K428 [32]. Sequences that are conserved which are good candidates for a role in CtBP2 nuclear localisation are the Pak1 phosphorylation site at Ser 164, given that the phosphorylation status of the corresponding site in CtBP1 (Ser158) regulates its subcellular localisation [22], and possibly the dimerisation domain. In the intact CtBP2 protein, the N-, C- and core domains do not function independently [31]. It is quite possible that whilst a.a. 120–445 are necessary for optimal nuclear localisation, a functional interaction between this region of the molecule and other sequences within a.a. 1–119 is required for a.a. 4–14-independent nuclear accumulation.

The experimental data obtained from our analysis of truncations and mutants of CtBP2 also provides additional insight into the mechanism of regulation of subcellular localisation by a.a. 4–14 containing the putative CtBP2 NLS. Zhao *et al *[27] demonstrated that this region does not, in fact, function as a classical NLS, but rather that it is necessary for lysines within it, primarily lysine 10, to be acetylated for it to direct localisation in the nucleus. Specifically they showed that lysines in this sequence are acetylated *in vivo *and that this is likely to be through the actions of the p300 acetyltransferase, a known CtBP binding protein. Their experiments using a non-acetylatable K10R mutant of CtBP2 showed that this acetylation is required for retention of CtBP2 in the nucleus, and that this mutant actually enhances CtBP2 nuclear export. Analogous to our experiments with the V72R mutant, they also showed that a different mutant in the PxDLS binding domain (A58E) does not affect acetylation by p300, and therefore p300 must bind to different sequences on CtBP2 than other PxDLS transcription factors. In contrast, Verger *et al *[28] concluded that this N-terminal lysine-arginine rich region functions as a classical nuclear localisation signal, with a role in nuclear import, rather than retention.

The experiments that we have performed do not distinguish between these two alternative mechanisms. Our finding that a.a 1–119 of CtBP2 is sufficient to drive efficient nuclear accumulation of EGFP provides an important advance in our understanding of the regions that regulate the subcellular distribution of CtBP2. However, the data are consistent with a role of a.a. 4–14 in either nuclear retention or import. Both previous studies demonstrated that the N terminal 25 amino acids cannot, alone, target a heterologous protein to the nucleus. This could be either due to an NLS or nuclear retention signal not being correctly presented to their target binding proteins in the context of these molecules, or the requirement for docking of acetyltransferases to a separate part of the molecule in order to achieve activation of the nuclear retention signal by acetylation. In CtBP2(1–119)-EGFP either the sequences may simply be sufficiently spaced from the EGFP for a.a. 4–14 to be correctly presented as an NLS, or this region may include the p300 binding site, allowing activation of the nuclear retention signal by acetylation. CtBP2(1–119)-EGFP is small enough to enter the nucleus by passive diffusion, and therefore the presence of a nuclear retention signal would be sufficient for nuclear accumulation. Expression of a.a. 1–119 in the context of a fusion with 2XEGFP would generate a larger protein that could only accumulate in the nucleus if it were actively imported through nuclear pores. However further experimentation would be required to determine conclusively whether this was due to a.a. 4–14 functioning as an NLS, or interaction of a.a. 1–119 with other actively imported proteins such as PxDLS containing transcription factors [23], HDM2 [12], or possibly p300 acetyltransferase [33].

Finally, we have identified heterodimerisation with CtBP2 as a novel mechanism that can promote the nuclear localisation of CtBP1. This interplay between the two proteins has also, very recently, been demonstrated by other investigators [28]. It will be interesting to determine the extent to which this heterodimerisation contributes to CtBP1 subcellular distribution in different cell types, compared with the other mechanisms that have been described previously. It is important to note that CtBP2 expression is clearly not an absolute requirement for nuclear CtBP1 activity in many cell types [34]. The contrasting subcellular localisations of over-expressed CtBP1 and CtBP2 in Cos-7 cells add weight to the growing argument that the two proteins are regulated differently. Indeed, studies on the role of CtBP1 and CtBP2 during murine development revealed a more severe and lethal phenotype in *Ctbp2*^-/- ^mice compared to *Ctbp1*^-/- ^mice [35]. This has been attributed to temporal and spatial differences in the expression of *Ctbp1 and Ctbp2 *during development [35]. Alternatively, it could be explained by the different modes of regulation of protein localisation and function between these two proteins, implying that perturbation of the constitutive nuclear function of CtBPs is responsible for the embryonic lethality of C*tbp2*^-/- ^animals. CtBP2 with an N-terminal motif that promotes its nuclear localisation is present in mice, man and fish. It is not yet known whether the smaller KRSR sequence in *Drosophila *CtBP is functional, and *Xenopus *CtBP does not contain any such motif in its N-terminus. Therefore, CtBP in *Xenopus*, and possibly *Drosophila*, will likely be dependent upon other protein-protein interactions for its recruitment to the nucleus. It is tempting to speculate, therefore, that this is an indicator of an increased importance of the nuclear activities of CtBP proteins in the regulation of the complex patterns of gene expression in higher organisms.

## Conclusion

CtBP1 and CtBP2 show a high degree of similarity at the sequence and functional level. Differential control of their subcellular localisation is likely to provide mechanisms to regulate critical nuclear and cytoplasmic functions of CtBPs in the cells of higher organisms. Here we have identified distinct regions in CtBP2 that play a key role in regulating the subcellular distribution of both CtBP2 and CtBP1 proteins.

## Methods

### Expression constructs

pcDNA3.1CtBP2(1–445)mh is previously described [12]. CtBP2(1–445)-EGFP was generated by ligation of a *Nhe*I/*Kpn*I digested insert of pcDNA3.1CtBP2(1–445)mh, into *Nhe*I/*Kpn*I-digested pEGFP-N1 (Clontech), followed by site directed mutagenesis (SDM; QuikChange, Stratagene), to place the EGFP in frame (forward primer 5'-CCCAACGAGCAGGTACCGCG-3'). Full-length pcDNA3.1CtBP1(1–440)mh was constructed by PCR amplification of *CTBP1 *cDNA, using the following primers: forward 5'-GCCGGAATTCATGGGCAGCTCGCACTTGCTC-3'; reverse 5'-GCGCCAAGCTTCAACTGGTCACTGGCGTGGTC-3', and ligation into the *Eco*RI/*Hind*III sites of pcDNA3.1(-)/Myc-HisA (Invitrogen). CtBP2(1–119)-EGFP was generated by *Nhe*I/*Sac*I digestion of pcDNA3.1CtBP2(1–445)mh and ligation into pEGFP-N1. Deletion mutants lacking residues 4–14 (CtBP2(1–445)Δ4–14 and CtBP2(1–119)Δ4–14) were generated by SDM in both pEGFP-N1 and pcDNA3.1(-)/Myc-HisA backgrounds (forward primer: 5'-TCCATGGCCCTTGGTCTCGACAGAATTTGT-3'), which replaced a.a. 4–14 with gly-leu (a *Bsa*I restriction site). CtBP2(1–119)NLS-EGFP was generated by replacing residues 4–14 with an SV40 large tumour antigen NLS (PKKKRV) (forward SDM primer: 5'-TCCATGGCCCTTCCGAAGAAGAAGCGGGTGGAGCTCGACAGAATTTGT-3'). PxDLS-binding mutants were constructed by SDM (forward primer: 5'-GGAAATCCACGAGAAGCGTCTAAACGAAGCCGT-3'). All clones were confirmed by sequence analysis.

### Cell culture and transfection

All cells were maintained in Dulbecco's modified Eagle medium (Invitrogen), supplemented with 10% foetal bovine serum (Autogen Bioclear) and penicillin (100 U/ml), streptomycin (100 μg/ml) and L-glutamine (292 μg/ml) (Invitrogen). Cells were seeded at the required density on glass coverslips 24 h prior to transfection. Effectene transfection reagent (Qiagen) was used to transfect 0.2 μg (0.4 μg for dual localisations) DNA into cells, as per the manufacturer's instructions. Cells were incubated for 48 h, and fixed for fluorescence microscopy.

### Immunofluorescence analysis

Cells were fixed with 4% paraformaldehyde in phosphate-buffered saline (PBS) for 10 min, and permeabilised using 0.1% Triton X-100/PBS for 20 min. They were blocked for 30 min in 3% bovine serum albumin (BSA)/PBS, and incubated as required with primary antibody (Anti-6xHis antibody, Abcam) for 1 h in 0.6% BSA/PBS, followed by Alexa594-conjugated species-specific secondary antibody (Molecular Probes). Cells were counterstained with 1 μg/ml DAPI during the secondary antibody incubation. When EGFP-fusion proteins only were visualised, all antibody incubation steps were omitted, and fixed and permeabilised cells were incubated with DAPI in 3% BSA/PBS for 10 min. Coverslips were mounted on slides with fluorescent mounting media (DakoCytomation). Cells were visualised using a Zeiss Axiovert 200 fluorescent microscope and images were collected using an Orca-ER digital camera (Hamamatsu), and processed using Openlab 3.5.1 Software (Improvision).

## Authors' contributions

LMB undertook experimental work and analysis, direct project supervision, and co-wrote the manuscript; LM undertook the majority of the cell localisation experiments, MD generated the majority of the vector reagents, SCG and AHM performed initial studies that lead to the identification of the NLS, JPB designed the project, provided overall supervision, and co-wrote the manuscript. All authors have read and approved the final manuscript.

## References

[B1] Boyd JM, Subramanian T, Schaeper U, La Regina M, Bayley S, Chinnadurai G (1993). A region in the C-terminus of adenovirus 2/5 E1a protein is required for association with a cellular phosphoprotein and important for the negative modulation of T24-ras mediated transformation, tumorigenesis and metastasis. Embo J.

[B2] Shi Y, Sawada J, Sui G, Affar el B, Whetstine JR, Lan F, Ogawa H, Luke MP, Nakatani Y (2003). Coordinated histone modifications mediated by a CtBP co-repressor complex. Nature.

[B3] Koipally J, Georgopoulos K (2000). Ikaros interactions with CtBP reveal a repression mechanism that is independent of histone deacetylase activity. J Biol Chem.

[B4] Sewalt RG, Gunster MJ, van der Vlag J, Satijn DP, Otte AP (1999). C-Terminal binding protein is a transcriptional repressor that interacts with a specific class of vertebrate Polycomb proteins. Mol Cell Biol.

[B5] Kim JH, Cho EJ, Kim ST, Youn HD (2005). CtBP represses p300-mediated transcriptional activation by direct association with its bromodomain. Nat Struct Mol Biol.

[B6] Hidalgo Carcedo C, Bonazzi M, Spano S, Turacchio G, Colanzi A, Luini A, Corda D (2004). Mitotic Golgi partitioning is driven by the membrane-fissioning protein CtBP3/BARS. Science.

[B7] Bonazzi M, Spano S, Turacchio G, Cericola C, Valente C, Colanzi A, Kweon HS, Hsu VW, Polishchuck EV, Polishchuck RS, Sallese M, Pulvirenti T, Corda D, Luini A (2005). CtBP3/BARS drives membrane fission in dynamin-independent transport pathways. Nat Cell Biol.

[B8] Spyer M, Allday MJ (2006). The Transcriptional Co-Repressor C-Terminal Binding Protein (CtBP) Associates with Centrosomes During Mitosis. Cell Cycle.

[B9] Schaeper U, Boyd JM, Verma S, Uhlmann E, Subramanian T, Chinnadurai G (1995). Molecular cloning and characterization of a cellular phosphoprotein that interacts with a conserved C-terminal domain of adenovirus E1A involved in negative modulation of oncogenic transformation. Proc Natl Acad Sci U S A.

[B10] Subramanian T, La Regina M, Chinnadurai G (1989). Enhanced ras oncogene mediated cell transformation and tumorigenesis by adenovirus 2 mutants lacking the C-terminal region of E1a protein. Oncogene.

[B11] Izutsu K, Kurokawa M, Imai Y, Maki K, Mitani K, Hirai H (2001). The corepressor CtBP interacts with Evi-1 to repress transforming growth factor beta signaling. Blood.

[B12] Mirnezami AH, Campbell SJ, Darley M, Primrose JN, Johnson PW, Blaydes JP (2003). Hdm2 recruits a hypoxia-sensitive corepressor to negatively regulate p53-dependent transcription. Curr Biol.

[B13] Lin X, Liang YY, Sun B, Liang M, Shi Y, Brunicardi FC, Feng XH (2003). Smad6 recruits transcription corepressor CtBP to repress bone morphogenetic protein-induced transcription. Mol Cell Biol.

[B14] Hamada F, Bienz M (2004). The APC tumor suppressor binds to C-terminal binding protein to divert nuclear beta-catenin from TCF. Dev Cell.

[B15] Meloni AR, Smith EJ, Nevins JR (1999). A mechanism for Rb/p130-mediated transcription repression involving recruitment of the CtBP corepressor. Proc Natl Acad Sci U S A.

[B16] Zhang Q, Yoshimatsu Y, Hildebrand J, Frisch SM, Goodman RH (2003). Homeodomain interacting protein kinase 2 promotes apoptosis by downregulating the transcriptional corepressor CtBP. Cell.

[B17] Bergman LM, Blaydes JP (2006). C-terminal binding proteins: Emerging roles in cell survival and tumorigenesis. Apoptosis.

[B18] Katsanis N, Fisher EM (1998). A novel C-terminal binding protein (CTBP2) is closely related to CTBP1, an adenovirus E1A-binding protein, and maps to human chromosome 21q21.3. Genomics.

[B19] Schmitz F, Konigstorfer A, Sudhof TC (2000). RIBEYE, a component of synaptic ribbons: a protein's journey through evolution provides insight into synaptic ribbon function. Neuron.

[B20] Spano S, Silletta MG, Colanzi A, Alberti S, Fiucci G, Valente C, Fusella A, Salmona M, Mironov A, Luini A, Corda D (1999). Molecular cloning and functional characterization of brefeldin A-ADP-ribosylated substrate. A novel protein involved in the maintenance of the Golgi structure. J Biol Chem.

[B21] Chinnadurai G (2002). CtBP, an unconventional transcriptional corepressor in development and oncogenesis. Mol Cell.

[B22] Barnes CJ, Vadlamudi RK, Mishra SK, Jacobson RH, Li F, Kumar R (2003). Functional inactivation of a transcriptional corepressor by a signaling kinase. Nat Struct Biol.

[B23] Criqui-Filipe P, Ducret C, Maira SM, Wasylyk B (1999). Net, a negative Ras-switchable TCF, contains a second inhibition domain, the CID, that mediates repression through interactions with CtBP and de-acetylation. Embo J.

[B24] Deltour S, Pinte S, Guerardel C, Wasylyk B, Leprince D (2002). The human candidate tumor suppressor gene HIC1 recruits CtBP through a degenerate GLDLSKK motif. Mol Cell Biol.

[B25] Riefler GM, Firestein BL (2001). Binding of neuronal nitric-oxide synthase (nNOS) to carboxyl-terminal-binding protein (CtBP) changes the localization of CtBP from the nucleus to the cytosol: a novel function for targeting by the PDZ domain of nNOS. J Biol Chem.

[B26] Lin X, Sun B, Liang M, Liang YY, Gast A, Hildebrand J, Brunicardi FC, Melchior F, Feng XH (2003). Opposed regulation of corepressor CtBP by SUMOylation and PDZ binding. Mol Cell.

[B27] Zhao LJ, Subramanian T, Zhou Y, Chinnadurai G (2006). Acetylation by p300 Regulates Nuclear Localization and Function of the Transcriptional Corepressor CtBP2. J Biol Chem.

[B28] Verger A, Quinlan KG, Crofts LA, Spano S, Corda D, Kable EP, Braet F, Crossley M (2006). Mechanisms Directing the Nuclear Localization of the CtBP Family Proteins. Mol Cell Biol.

[B29] PredictNLS server. http://cubic.bioc.columbia.edu/predictNLS/.

[B30] Alpatov R, Munguba GC, Caton P, Joo JH, Shi Y, Hunt ME, Sugrue SP (2004). Nuclear speckle-associated protein Pnn/DRS binds to the transcriptional corepressor CtBP and relieves CtBP-mediated repression of the E-cadherin gene. Mol Cell Biol.

[B31] Nardini M, Spano S, Cericola C, Pesce A, Massaro A, Millo E, Luini A, Corda D, Bolognesi M (2003). CtBP/BARS: a dual-function protein involved in transcription co-repression and Golgi membrane fission. Embo J.

[B32] Kagey MH, Melhuish TA, Wotton D (2003). The polycomb protein Pc2 is a SUMO E3. Cell.

[B33] Zhao LJ, Subramanian T, Zhou Y, Chinnadurai G (2005). Acetylation by p300 regulates nuclear localization and function of the transcriptional corepressor CtBP2. J Biol Chem.

[B34] Grooteclaes M, Deveraux Q, Hildebrand J, Zhang Q, Goodman RH, Frisch SM (2003). C-terminal-binding protein corepresses epithelial and proapoptotic gene expression programs. Proc Natl Acad Sci U S A.

[B35] Hildebrand JD, Soriano P (2002). Overlapping and unique roles for C-terminal binding protein 1 (CtBP1) and CtBP2 during mouse development. Mol Cell Biol.

[B36] Wan L, Almers W, Chen W (2005). Two ribeye genes in teleosts: the role of Ribeye in ribbon formation and bipolar cell development. J Neurosci.

